# Comparison of the effects of ketamine and fentanyl-midazolam-medetomidine for sedation of rhesus macaques (*Macaca mulatta*)

**DOI:** 10.1186/s12917-016-0721-9

**Published:** 2016-06-08

**Authors:** Henri G. M. J. Bertrand, Yvette C. Ellen, Stevie O’Keefe, Paul A. Flecknell

**Affiliations:** Comparative Biology Centre, Newcastle University, Framlington Place, Newcastle Upon Tyne, NE2 4HH UK; Faculty of Veterinary Medicine, University of Liège, Boulevard de Colonster, Liège, 4000 Belgium; School of Veterinary Medicine and Science, University of Nottingham, Sutton Bonington Campus, Loughborough, LE12 5RD UK; Institute of Neuroscience, Newcastle University, Newcastle Upon Tyne, NE1 7RU UK

**Keywords:** Sedation, Ketamine, Fentanyl, Midazolam, Medetomidine, Recovery macaque

## Abstract

**Background:**

This study assessed the effects of sedation using a combination of fentanyl, midazolam and medetomidine in comparison to ketamine. Rhesus Macaques (*Macaca mulatta*), (*n* = 16, 5 males and 3 females randomly allocated to each treatment group) received either ketamine (KET) (10 mg.kg^−1^) or fentanyl-midazolam-medetomidine (FMM) (10 μg/kg^−1^; 0.5 mg.kg^−1^; 20 μg.kg^−1^) both IM. Oxygen (100 %) was provided by mask and heart rate, blood pressure, respiratory rate, EtCO_2_ and depth of sedation were assessed every 5 min for 20 min. After the last time point, FMM monkeys were reversed with atipamezole-naloxone (0.2 mg.kg^−1^; 10 μg.kg^−1^). Recovery was scored using clinical scoring scheme. Differences in physiological parameters and quality of sedation were compared using Area Under the Curve (AUC) method and either Mann-Witney or t-student tests.

**Results:**

Heart rate (beats/min) (Ket = 119 ± 18; FMM = 89 ± 17; *p = 0.0066*), systolic blood pressure (mmHg) (Ket = 109 ± 10; FMM = 97 ± 10; *p = 0.0313)*, and respiratory rate (breaths/min) (Ket = 39 ± 9; FMM = 29 ± 10; *p = 0.0416*) were significantly lower in the FMM group. End-tidal CO_2_ (mmHg) did not differ between the groups (KET = 33 ± 8; FMM = 42 ± 11; *p = 0.0462*)_._ Although some depression of physiological parameters was seen with FMM, the variables all remained within the normal ranges in both groups. Onset of a sufficient degree of sedation for safe handling was more rapid with ketamine (KET = 2.9 ± 1.4 min; FMM = 7.9 ± 1.2 min; *p = 0.0009*), but FMM recovery was faster (KET = 21.4 ± 13.4 min; FMM = 9.1 ± 3.6 min; *p = 0.0379*) and of better quality (KET = 1.3 ± 0.9; FMM = 7.4 ± 1.9; *p = 0.0009)* most probably because of the effectiveness of the reversal agents used.

**Conclusion:**

FMM provides an easily reversible immobilization with a rapid and good recovery quality and may prove a useful alternative to ketamine.

## Background

Ketamine, a dissociative anaesthetic, is widely used for the chemical immobilization of non–human primates (NHPs). When administered alone it induces a cataleptic state allowing safe handling as the biting reflex is inhibited [1, 2]. Protective airway reflexes are conserved and voluntary movement can still occur. Ketamine has some analgesic activity, allowing minor procedures such as skin suturing to be undertaken but it is not suitable as a sole agent for more invasive procedures. Ketamine is an NDMA receptor antagonist, and so should prevent “wind-up” associated with noxious stimuli [3], but this effect has never been assessed in NHPs. Several side effects have been described in primates and other species, including pain on injection, muscular and nerve damage at the site of injection [2, 4–6], and rarely, seizures [7–10]. Recovery delirium also occurs but this can be reduced by addition of other agents. Despite these problems, ketamine is widely used in primates, primarily because of its good safety profile and relative lack of depression of the cardiovascular and respiratory systems. For some procedures, a greater degree of analgesia and muscle relaxation would be advantageous. Although this can be achieved by the addition of medetomidine, as in other species, recovery is still relatively prolonged even after reversal of the medetomidine with atipamezole [11]. Replacement of ketamine with agents that have less prolonged depressant effects, or are reversible with specific antagonists could therefore be advantageous. In human medicine, the combination of fentanyl and midazolam has been widely used for conscious sedation for minor procedures, and at higher dose rates for surgery [12–15]. Combinations of opioids and benzodiazepines seem to be less effective in Rhesus macaques [16], however in other species, addition of medetomidine to these combinations produces fully reversible anaesthesia [17–19] and an initial report suggests this combination can be used successfully in non-human primates [20].

This study compared the effects of a combination of fentanyl-midazolam-medetomidine (FMM), followed by reversal with naloxone and atipamezole, with ketamine (KET) in rhesus macaques.

## Results

### Sedation procedure

One female from the KET group started to recover 10 min after administration of the anaesthetic and was placed in a recovery cage. No physiological parameters were recorded after this time point, but the recovery time and the assessment of the recovery quality were assessed. All of the remaining primates were successfully immobilized after the administration of either KET or FMM.

### Onset of sedation and recovery times

**Fig. 1:**
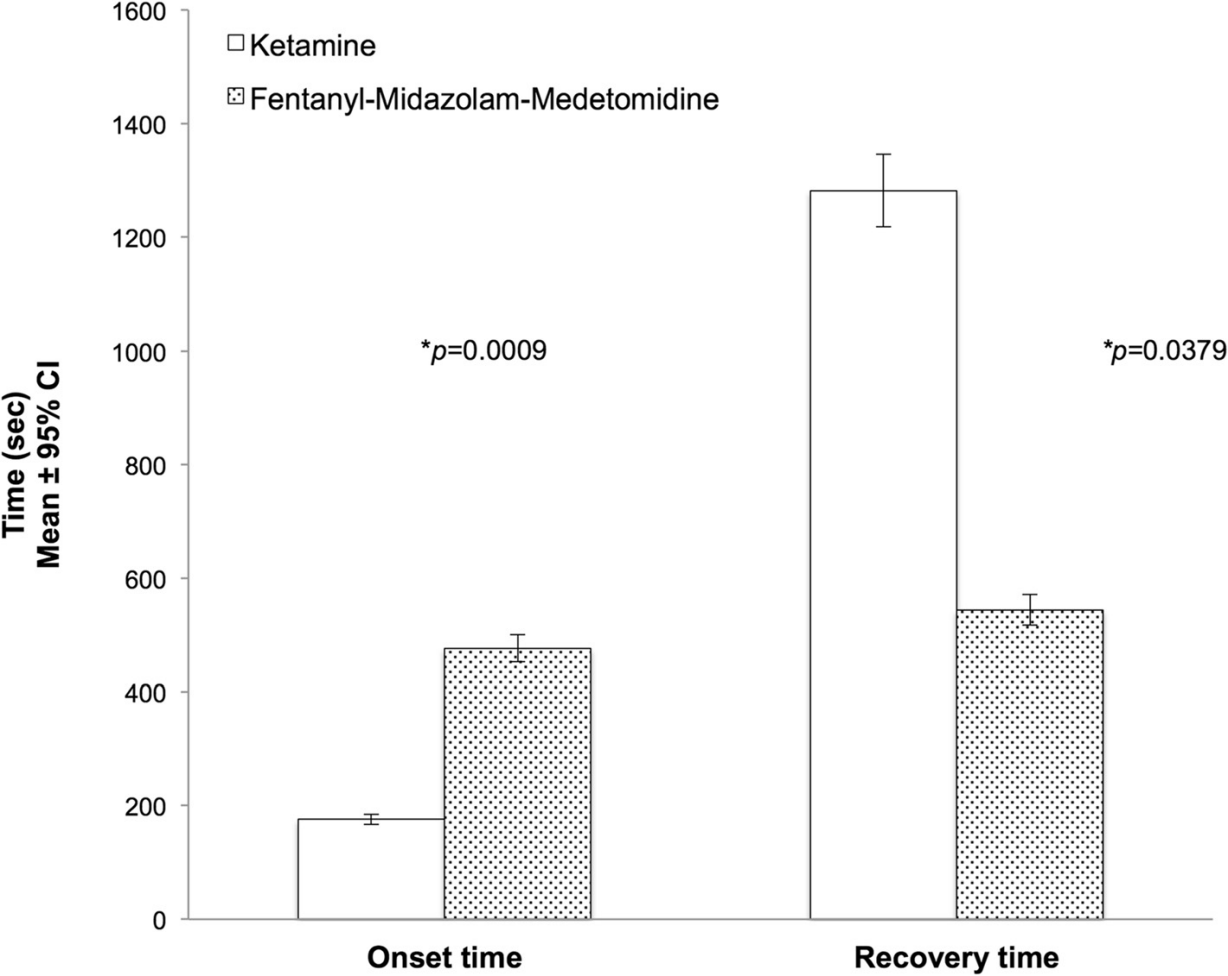
Onset of sedation and Recovery times in Rhesus macaques receiving ketamine (*n* = 8) or fentanyl-midazolam-medetomidine (*n* = 8) Induction times: Ketamine (KET) 2.9 ± 1.4 min; Fentanyl-midazolam-medetomidine (FMM) 7.9 ± 1.2 min. Recovery times: Ketamine (KET) 21.4 ± 13.4 min; Fentanyl-midazolam-medetomidine (FMM) 9.1 ± 3.6 min. An asterisk next to a *p*-value(*p) indicates a significantly differences

The onset of a sufficient degree of sedation for safe handling was significantly shorter in the KET group (2.9 ± 1.4 min) than in the FMM group (7.9 ± 1.2 min*)(p =* 0.0009). Recovery was significantly faster in the FMM group (9.1 ± 3.6 min) compared to the KET group (21.4 ± 13.4 min) *(p =* 0.0379) (Fig. 1).

### Physiological parameters and depth of sedation

The physiological data are summarised in Table 1. Area under the curves (AUCs) comparisons showed significant differences between the two treatment groups. The heart rate (HR) *(p =* 0.0066), Respiration rate (RR) *(p =* 0.0416), systolic blood pressure (BPsyst) *(p =* 0.0313) and the end-tidal CO_2_ (EtCO_2)_*(p =* 0.0462) AUCs were significantly lower in the FMM group. However, no significant difference was found in SpO_2_ between the two groups (*p* > 0.1). The overall degree of sedation over the 20 min procedure was deeper in the FMM group with a significantly higher score *(p =* 0.0009) than the KET group.

**Table 1:** Physiological parameters recorded over 20 min for each treatment group

	Treatment groups										*p-value*
	Ketamine					Fentanyl-Midazolam-Fentanyl					
Time	0 *n = 8*	5 *n = 8*	10 *n = 8*	15 *n = 7*	20 *n = 7*	0 *n = 8*	5 *n = 8*	10 *n = 8*	15 *n = 8*	20 *n = 8*	
HR*(beat/min)*	134 ± 17	121 ± 18	117 ± 17	110 ± 14	112 ± 18	97 ± 17	90 ± 17	87 ± 18	86 ± 18	85 ± 17	0.0066*
SpO_2_ *(%)*	98.2 ± 1.0	99.8 ± 0.7	100 ± 0	99.9 ± 0.4	100 ± 0	96.1 ± 7.8	99.6 ± 0.7	99.8 ± 0.5	99.8 ± 0.7	99.6 ± 0.7	0.7254
RR*(breaths/min)*	36 ± 8	41 ± 8	36 ± 6	40 ± 11	43 ± 11	30 ± 10	28 ± 12	28 ± 11	29 ± 11	29 ± 10	0.0416*
EtCO_2_ *(mmHg)*	36 ± 8	32 ± 9	32 ± 9	34 ± 9	32 ± 8	39 ± 12	44 ± 11	43 ± 11	44 ± 10	42 ± 10	0.0462*
BPsyst*(mmHg)*	111 ± 10	111 ± 9	107 ± 9	107 ± 11	108 ± 13	101 ± 9	102 ± 10	97 ± 10	93 ± 9	90 ± 8	0.0313*
SD	12 ± 3	12 ± 2	11 ± 3	11 ± 3	11 ± 4	17 ± 1	18 ± 1	18 ± 1	18 ± 1	18 ± 1	0.0009*

Data are shown as mean ± 1 SD. *n* represents the number of animals that provide data at the each time point. In the ketamine group one of the primates started to recover after 10 min of sedation and so recording session was stopped. Areas Under the Curve (AUCs) were calculated for each parameter. The Shapiro-wilk test showed a normal distribution for Heart Rate (HR), the Respiration Rate (RR), the Systolic Blood Pressure (BPsyst) and the end-tidal CO_2_ (EtCO_2_). These AUCs parameters were compared with Student t test. The oxygen saturation (SpO_2_) and the sedation depth (SD) were compared with the Mann–Whitney U test. An asterisk next to the *p-value* indicates a significant statistical difference at α threshold of 5 %

### Recovery quality

**Fig. 2:**
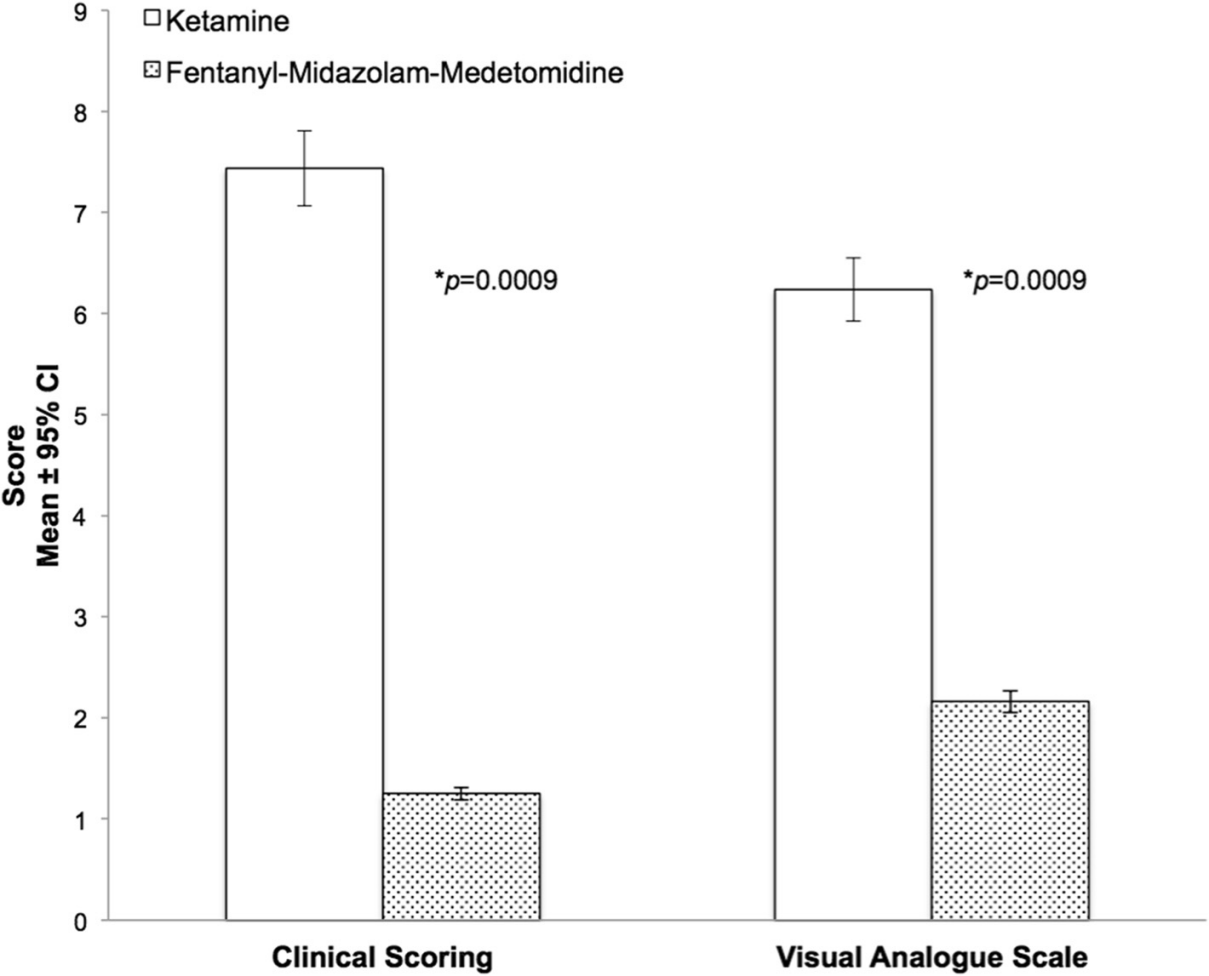
Recovery quality results in Rhesus macaques receiving ketamine (*n* = 8) or fentanyl-midazolam-medetomidine (*n* = 8). The histograms represent the mean ± 95 % of confidence interval. The results are expressed as mean ± 1 SD. RCS: Ketamine 7 ± 2; Fentanyl-midazolam-medetomidine 1 ± 1. VAS: Ketamine 6.2 ± 0.8; Fentanyl-midazolam-medetomidine 2.2 ± 0.5. An asterisk next to a *p*-value(*p) indicates a significantly differences

**Fig. 3:**
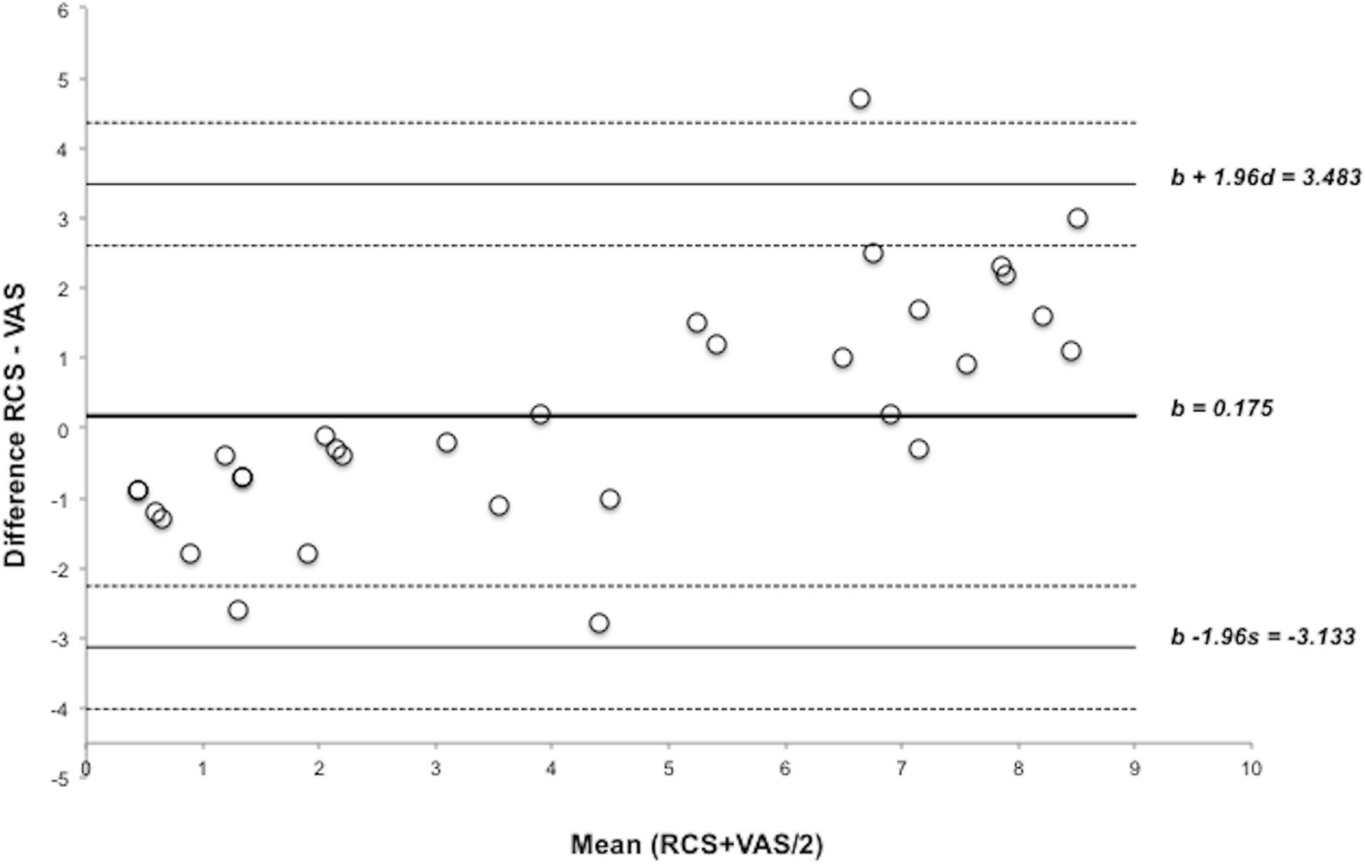
Bland-Altman plots comparing the quality of recovery in Rhesus macaques receiving ketamine (*n* = 8) or fentanyl-midazolam-medetomidine (*n* = 8). The differences between the scores for each method are plotted against the mean of the two methods. (*b*) = bias; (*b + 1.96 s; b – 1.96 s*) = Agreement limits. The shaded lines represent the 95 % confidence intervals of the agreement limits

Recovery quality was significantly better in the FMM group after administration of reversal agents, than in the ketamine group, as assessed by both the visual analogue scale (VAS) and the recovery clinical scoring scheme (RCS) (Fig. 2). The Bland-Altman plots demonstrated a non-significant bias of 0.1437 and a reasonably good level of agreement between the two tests (Fig. 3). However it also showed a non-homogeneous distribution of the plots that was probably due to the scale difference between the VAS and the RCS. For the Kappa statistic analysis, the average of the two observers for each subject was used. The comparison of recovery group results gave a kappa statistic k = 0.662 with a significance of *p* = 0.01. According to usual interpretation of this result [21], there was a substantial agreement between the RCS and VAS.

## Discussion

In this study the administration of the FMM combination to rhesus macaques provided a deeper and more reliable sedation and a better recovery score than ketamine sedation. The impacts of the two regimens on physiological parameters were consistent with the known properties and mechanism of action of the agents used.

High ketamine liposolubility results in rapid bioavailability in the central nervous system, resulting in rapid onset of action [22]. This rapid onset of sedation was observed in the present study and is similar to that reported in previous studies [23–25]. Attainment of sedation sufficient for safe handling was significantly shorter following administration of ketamine than following FMM, likely due to the different rates of absorption and distribution of the components of the combination. The onset of a sufficient degree of sedation to allow safe handling after administration of FMM observed in this study was longer than that reported by Votava *et al.*, using a comparable sedation protocol [20]. This difference may be due to differences in the doses of the agents used in the two studies and the use of hyaluronidase to speed absorption in Votava study. Recovery time in the FMM group was significantly shorter than the KET group, very likely due to the use of specific antagonists to reverse two of the components used for sedation [26, 27]. We chose not to administer flumazenil to reverse midazolam, since our experience has shown that midazolam has minimal sedative effects at the dose used in this species. In this study, because no post-procedural pain was anticipated, naloxone was used to reverse the effects of fentanyl. As an alternative, butorphanol, nalbuphine and buprenorphine have all been shown to be effective antagonists [28–30]. This latter approach has the advantage of providing continued analgesia following reversal of the fentanyl, because of administration of agents with either k agonist/μ antagonist activity, or partial μ agonist actions. A duration of sedation prior to reversal of the FMM regimen of 20 min was chosen as it represented the mean time that NHPs can be safely handled under ketamine sedation [24, 25].

Anaesthetic agents decrease central nervous system activity and the assessment of somatic withdrawal reflexes is generally accepted as a means of assessing anaesthetic depth [3]. Due to its mechanism of action, ketamine produces a unique cataleptic state, but one which can be considered as light sedation based on the degree of suppression of reflex responses (eg withdrawal reflexes) [3, 4]. Used alone, ketamine increases muscular tone in many species [31–33] but this effect is not as marked in non human primates as in other species [24]. Seizures [7–10] and tissue necrosis [5, 6, 34] can also occur with ketamine injection. In this study, none of these complications occurred, however one of the female primates only remained in lateral recumbency for less than ten minutes post-injection and this has been observed in other studies [24, 25]. Several hypotheses such as the variation of the injection site with incomplete intramuscular administration, individual variability or a possible acquired tolerance to ketamine [25] can explain this observation. In this study, the FMM protocol provided a deeper sedation, comparable to surgical anaesthesia. Following completion of this study, we have used the combination to enable intubation as jaw tone is markedly reduced and laryngeal reflexes absent. We have also repaired deep and superficial wounds, which resulted from fights between cage-mates.

Pulse oximetry was used successfully to assess oxygen saturation, but this technique can be compromised by poor peripheral tissue perfusion [2], such as can occur when alpha_2_ agonists are administered. This was not a problem in the current study. Systolic blood pressure was measured by a non-invasive oscillometric method. The cuff used to measure the blood pressure followed the general recommendation that the cuff width should equal 40 % of the limb circumference [35]. However, this method has been shown to underestimate systolic and diastolic blood pressures by between 5 and 20 mmHg depending on the cuff location [36–38]. Adequacy of ventilation was assessed using capnography, since this allowed assessment of respiratory rate and pattern and the EtCO_2_. The EtCO_2_ is usually measured at the distal extremity of the endo-tracheal tube [39, 40], but in the present study, the EtCO_2_ was measured in the ventral meatus of the nose with a sample line linked to a side stream capnography device. This approach provided a good waveform and is a technique that could be used routinely for anaesthetic monitoring in this and other primate species

However validation of the approach should be performed as published data comparing the technique are available only in people [41–44].

The significant differences in cardiovascular and respiratory parameters between the sedation protocols in this study are consistent with the mechanism of action of the agents used. Ketamine has a minimal impact on the respiratory system with minimal modification of the ventilation parameters [3]. The breathing rate observed and EtCO_2_ were similar to those described in a previous study in rhesus macaques but the EtCO_2_ values recorded were lower [23, 45]. Ketamine affects the cardiovascular system by stimulating the sympathetic pathway and increasing circulating catecholamine concentrations [3]. Heart rate and systolic blood pressure effects were also consistent with the literature [20, 23, 45, 46]. Due to the known mechanisms and interactions of the individual components of the regimen, it was expected the FMM protocol would have more negative influences on cardiovascular and respiratory functions. Midazolam, has sedative and anxiolytic effects but minimal effects on cardio-respiratory physiology [3, 4], however medetomidine and fentanyl both depress these system and their effects are potentiated when they are combined [3]. Although FMM produced a significant decrease of heart rate, systolic blood pressure and respiratory function, these values stayed within acceptable ranges for deeply sedated primates [1, 3]. There appears to be no previously published information on the impact of fentanyl-midazolam-medetomidine on these parameters in macaques, but the bradycardia noted in this study was similar to that reported by Votava *et al.* [20]. This combination has varying effects in other species. In rats, rabbits, Mongolian gerbils and chinchillas, bradycardia is a consistent finding, whereas effects on blood pressure varied. In contrast to other species in which moderate hypotension was reported, rats developed moderate hypertension [17].

The quality of the recovery was assessed by two methods, a clinical scoring scheme (RCS) and a visual analogue scale (VAS). The VAS is a subjective single-item test that in this study was used only to assess the quality of recovery. VAS assessments are widely used to assess pain [47], anxiety [48] and other items [49, 50]. Such systems have the advantage of being quick and easy to perform and also to have good reliability [51]. The clinical scoring scheme used in this study was the first attempt to construct and use a multiple-item test to assess recovery in non-human primates. The scale used was based on the Pediatric Anesthesia Emergence Delirium Scale (PAED) [52, 53]. The items chosen to build the RCS were based on their abilities to show modification in cases of excitation or poor recovery and on the facility to observe these modifications. The two methods of assessment demonstrated a strong correlation and a strong agreement with a non-significant bias. The video extracts, used for the scoring the quality of recovery, focused on the period around first attempt to sit. This period can be considered a critical point in recovery as excitation, ataxia or hallucination can lead to injury [3, 54]. The study results showed that the recovery quality was significantly better in the FMM treatment group. There are several possible explanations for this result. The use of specific antagonists in the FMM group may have reversed not only the sedative effects of the three components but also their side effects [26, 27]. The poor quality recovery in the ketamine group may have been the results of ketamine’s dissociative effects [3]. Previous studies in dogs and cats, using ketamine, did not report a difference in the quality of recovery compared to other anaesthetic protocols [55, 56]. However the administration of ketamine to children and horses prior the end of anaesthesia resulted in a decrease in the quality of recovery in comparison with the use of α_2_-agonists, benzodiazepine or acepromazine [57, 58].

## Conclusion

In conclusion, this study demonstrated the efficacy of the combination of fentanyl, medetomidine and midazolam to immobilise rhesus macaques. This regimen offers a potentially useful alternative to ketamine, since specific antagonists can rapidly reverse it, and this results in a better quality of recovery.

## Methods

### Animals

The animals were purpose bred for research in the UK, and were supplied from the Centre for Macaques to Newcastle University. Animals were housed in a Home Office (United Kingdom authority) accredited facility and in compliance with the Animal Scientific Procedure Act 1986 and the European Directive 2010/63/EU. Rhesus macaques aged from 3 to 10 years and weighing from 4 to 17.7 kg (n = 16, 10 males and 6 females) scheduled, in November 2014, for annual health checks and tuberculosis testing were used in this study. The animals were housed at Comparative Biology Centre (Newcastle University, United Kingdom), in indoor pens with a solid floor and windows allowing a view on the other pens and the corridors. A minimum floor area 4.40 m^2^ was provided for each animal or pair of animals. A smaller pen with a squeeze-back system was located between each housing pen. Enrichment devices and substrate for foraging were provided. Animals were maintained on a light:dark cycle of 12 h:12 h. at a temperature of 22 °C and with 15 air changes per hour and a relative humidity of 24 %. Primates were fed with adapted old world primate diet (Special Diets Service, Witham, United Kingdom) and received tap water *ad libitum* at the time of the study. Forage mix was provided daily to all animals (LBS Biotechnology, United Kingdom). Except for two males, all the primates were paired-housed. The single housing was due to exceptional circumstances unrelated to the research protocols. The primates had not been sedated or anaesthetised during the two weeks prior the start of the study.

### Sedation protocols

Animals (5 males and 3 females per group) were assigned randomly to receive either ketamine (KET) or Fentanyl-Midazolam-Medetomidine (FMM). Animals were fasted at least 4 h prior to the administration of the sedative agents. Both sedation protocols were administered by intramuscular injection in the femoral quadriceps, the femoral biceps or the gluteal maximus. Ketamine (Ketaset 100 mg/ml Solution for Injection, Zoetis, London, United Kingdom) was administered at the dose of 10 mg.kg^−1^ [23, 24]. For the FMM protocol, the dose each drug was determined based on a pilot study (data not reported here). Fentanyl (Fentanyl 50 micrograms/ml, Martindale Pharmaceutical, Romford, United Kingdom), midazolam (Midazolam 5 mg/ml, Hameln pharmaceutical ltd, Gloucester, United Kingdom) and medetomidine (Domitor 1 mg/ml, Vetoquinol UK ltd, Buckingham, United Kingdom) were administered at 10 μg.kg^−1^, 0.5 mg.kg^−1^ and 20 μg.kg^−1^, respectively. Due to the high injection volume 2 injection sites were used. Midazolam and medetomidine were mixed in the same syringe and injected separately from the fentanyl. When primates lost their righting reflex and could be safely handled, they were carried to a room outside of the NHP unit. At the end of the procedure, primates in the KET group were placed in a recovery cage and monitored until the return of the righting reflex. In the FMM group, naloxone at 10 μg.kg^−1^ (Naloxone 400 micrograms/ml Solution for injection/infusion, Hameln pharmaceutical ltd, Gloucester, United Kingdom) and atipamezole (Antisedan 5 mg/ml, Vetoquinol UK ltd, Buckingham, United Kingdom) at 0.22 mg.kg^−1^ were mixed in the same syringe and administered by intramuscular injection 20 min after the onset of sedation. Then primates were transferred to a recovery cage and monitored until the return of the righting reflex.

### Sedation support and monitoring

**Fig. 4:**
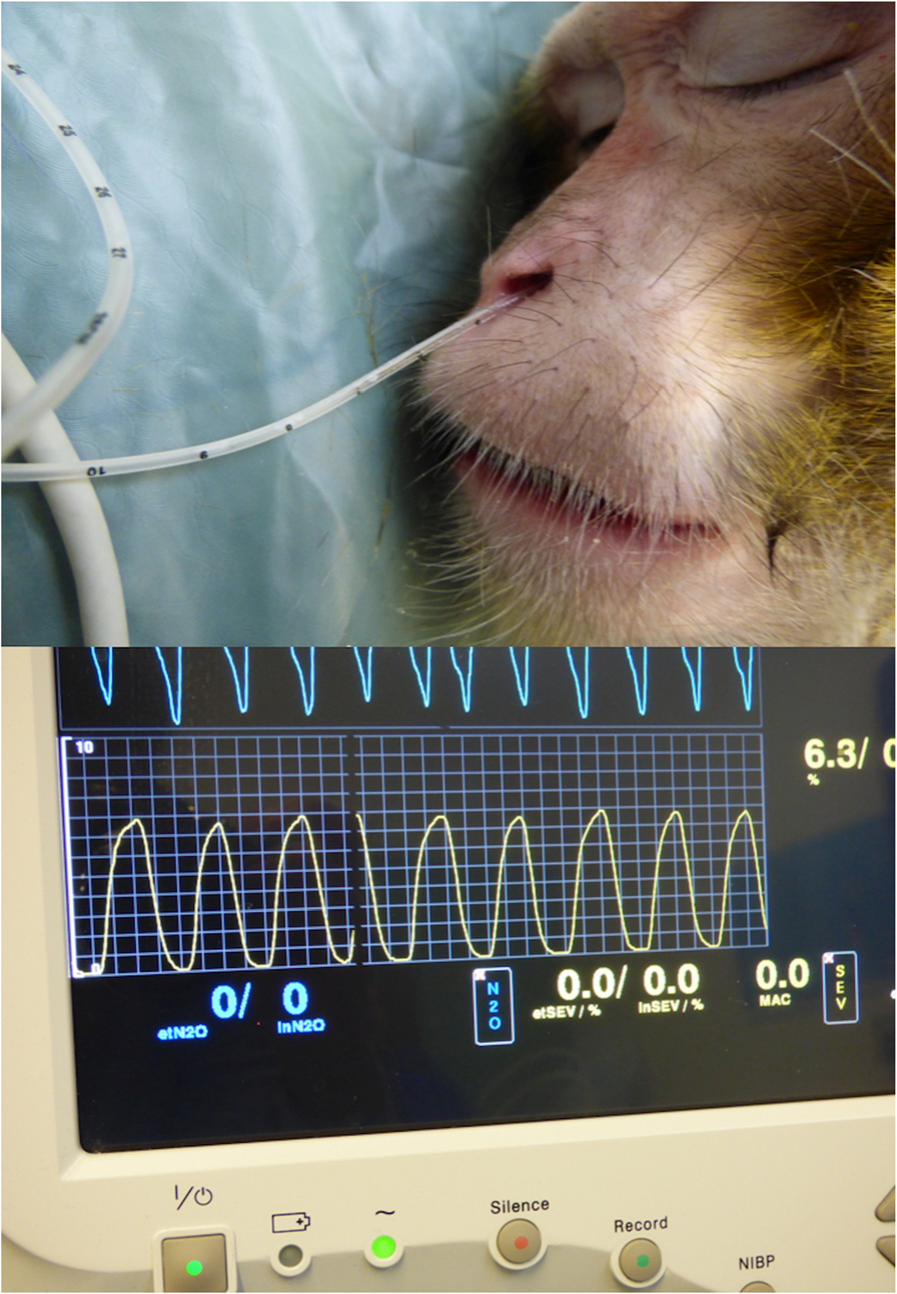
Respiration monitoring. A soft lubricated tube was inserted in the ventral meatus of one of the nostril. The tube was linked to a side-stream capnograph providing the visualization of a waveform and end-tidal CO_2_ value

Physiological parameters and depth of the sedation were measured every 5 min for 20 min procedure consisting of venous blood sampling, dental examination, an intradermal tuberculosis test and a complete physical examination. Animals were placed in lateral recumbency, covered with a forced-air warming blanket set at 38 °C (Bair hugger model 505, Augustine Medical, USA) and received 100 % oxygen supplementation using a face mask (2 to 4 litres per min). A Vitalogik 4500 monitoring system (Charter-Kontron Ltd, Milton Keynes, United Kingdom) was used to measure vital signs. Heart rate (HR) and oxygen saturation (SpO_2_) were assessed using an absorbance pulse oxymeter probe placed on a finger. Blood pressure was assessed using an oscillometric method with a blood pressure cuff (Critikon Dura-Cuf, GE Healthcare, Hatfield, United Kingdom) appropriate for the size of the animal. The cuff was placed on the opposite arm to that used for pulse oximetry to record the blood pressure from the brachial artery. Two measures were taken at each time point and the mean calculated. The respiration rate (RR) and the end-tidal CO_2_ (EtCO_2_)_,_ were measured by the side stream gas analyser integrated in the electronic monitoring system. The gas sampling rate was 50 ml/min. A soft neonatal feeding tube with 1.7 mm of diameter and 38 cm of length (100 % latex-free Premature Infant Feeding tube, Bard Ltd, Crawley, United Kingdom) attached to the gas sampling line was lubricated with lidocaine gel (Anbesol teething gel, Alliance pharmaceutical, Chippenham, United Kingdom) and gently advanced to a depth of 3 cm into the ventral meatus of one of the nostril (Fig. 4). This operation was repeated at each time point. The depth of the sedation was assessed by a clinical scoring scheme adapted from previous publications (Table 2).

**Table 2:** Sedation Depth Scoring System. A higher score indicates a deeper sedation

Score	Movement	Palpebral reflex	Jaw tone	Withdrawal reflex
1	Whole body	Blinking + others movement	increased	Normal
2	Limb/foot/Hand	Blinking	Normal	Weakly
3	Facial	Weak blinking	Decreased	Delayed
4	Twitching fingers	Delayed blinking	Minimal	Only digits movements
5	No	No	No	No

### Onset of sedation and recovery times

All of the sedation procedures were videotaped and analysed at a later date. This enabled blinding of the assessor to the treatment given. The time from administration of the sedative to the time at which it was considered that an animal that could be safely handled was recorded. The recovery time was the time between the end of the 20 min procedure and the return of the righting reflex. The end of the procedure was comparable with the injection of the antidote mix in the FMM group

### Recovery quality assessment

Samples of the video-recording from 2 min before to 2 min after the first attempt to sit were selected and the quality of recovery assessed by two treatment-blinded observers using two methods. A recovery clinical scoring scheme (RCS) consisting of a multiple-item list (Table 3) and a visual analogue scale (VAS), with a 10 cm line anchored with “best possible recovery” and “worst possible recovery” were used for this assessment. In both scoring schemes, a high score indicated a poor recovery. The observers were veterinary technicians with extensive experience of working with NHPs. Depending on the numeric result, each recovery was classified using the categories in Table 4.

**Table 3:** Recovery Clinical Scoring Scheme. System based on 15 points with the principle that a higher score indicates a poorer recovery

•Eyes					
Score	2	1	0	+1	+1
	Close	Semi-close	Open	Nystagmus	Rubbing its eyes
•Mouth					
Score	3	2	1	0	
	Vomitting	Lips smacking/Nausea sign	Hypersalivation	Nothing	
•Body position					
Score	3	2	1	0	
	Lateral/ventral recumbency	Unsuccessful attempt to sit	Successful attempt to sit/Sit but wobbly	Sit and stable	
•Ataxia					
Score	3	2	1	0	
	Strong	Mild	Slight	Absence	
•Environment awareness					
Score	2	1	0		
	No	Partially	Yes		
Total: /15

**Table 4:** Recovery quality category

Category	VAS	RCS
Good	0–3.33	0–5
Moderate	3.34–6.67	6–10
Bad	6.68–10	11–15

*VAS* visual analogue scale, the range are expressed in cm. *RCS* recovery clinical scoring, the ranges are expressed in arbitrary units

### Statistical analysis

For the HR, BPSyst, RR, EtCO2, SpO2 and sedation depth, Area Under the Curves (AUCs) were estimated using the trapezoidal method [59, 60]. The AUCs normality distribution was assessed using the Shapiro-Wilk test. Student’s t test was performed where data were normally distributed; otherwise the Mann–Whitney U test was used.

A Mann–Whitney U test was used to compare sedation times and recovery times between the two groups and the RCS and the VAS sedation recovery scores. Bland-Altman plots and Kappa statistic methods were used to assess the agreement between the VAS and RCS results.

Statistical analyses were performed with SPSS statistic software (vers. 22, IBM, USA) and Excel (vers. 14.3.0, Microsoft, USA). A *p*-value < 0.05 was considered statically significant

## Abbreviations

AUC, area under the curve; BPsyst, systolic blood pressure; EtCO_2_, endt-tidal CO_2_; FMM, Fentanyl-Midazolam-Medetomidine; HR, heart rate; IM, intramuscular; KET, ketamine; NHPs, non-human primates; PAED, pediatric anesthesia emergence delirium scale; RCS, Recovery Clinical scoring scheme; RR, respiration rate; VAS, visual analogue scale
